# Efficacy and safety of tranexamic acid in posterior lumbar interbody fusion: a meta-analysis of randomized controlled trials

**DOI:** 10.1186/s13018-022-03493-8

**Published:** 2023-01-05

**Authors:** Haopeng Luan, Kai Liu, Cong Peng, Qi Tian, Xinghua Song

**Affiliations:** 1 Department of Spine Surgery, The Six Affiliated Hospital of Xinjiang Medical University, Ürümqi, 830002 Xinjiang China; 2 Department of Trauma and Microreconstructive Surgery, The First Affiliated Hospital of Xinjiang Medical University, Ürümqi, 830054 Xinjiang China; 3 Department of Bone Tumor Surgery, The First Affiliated Hospital of Xinjiang Medical University, Ürümqi, 830054 Xinjiang China

**Keywords:** Tranexamic acid, Posterior lumbar interbody fusion, Blood loss, Meta-analysis

## Abstract

**Objective:**

To evaluate the efficacy and safety of tranexamic acid (TXA) in hemostasis in patients undergoing posterior lumbar interbody fusion (PLIF) by meta-analysis.

**Methods:**

This study was registered on the International Prospective Register of Systematic Reviews (PROSPERO) (ID: CRD42022354812). The databases PubMed, Cochrane Library, Web of Science, and Embase were searched for randomized controlled trial (RCT) papers on the use of TXA in patients with PLIF from database establishment to August 2022. Two researchers screened the literature, extracted data, evaluated the risk of bias of the included studies, recorded the authors, sample size, type of study design, and TXA dose of each study, and extracted the intraoperative blood loss, number of blood transfusions, total blood loss, drainage volume, operation time, and incidence of deep venous thrombosis in each study. Meta-analysis was performed using RevMan 5.4 software provided by Cochrane Library.

**Results:**

A total of 14 RCTs with a total of 1681 patients were included in this study, including 836 patients in the TXA group and 845 patients in the control group. The intraoperative blood loss [mean difference (MD) = − 125.97, 95% confidence interval (CI) (− 138.56, − 113.37), *P* < 0.0001] and less total blood loss [MD = − 204.28, 95% CI (− 227.38, − 181.18), *P* < 0.00001] in TXA group were lower than the control group. Statistical significance was also observed in postoperative drainage volume [MD = − 115.03, 95% CI (− 123.89, − 106.17), *P* < 0.00001], operation time [MD = − 8.10, 95% CI (− 14.49, − 1.71), *P* = 0.01], and blood transfusion rate [odds ratio (OR) = 0.30, 95% CI (0.23, 0.39), *P* < 0.00001]. However, there was no statistical difference observed in the incidence of deep venous thrombosis [OR = 0.83, 95% CI (0.56, 1.21), *P* = 0.33].

**Conclusion:**

The application of TXA in PLIF can reduce intraoperative blood loss, total blood loss, drainage volume, the incidence of transfusion events, and operation time without increasing the risk of deep venous thrombosis.

## Introduction

Patients diagnosed with degenerative lumbar spine diseases such as lumbar spinal stenosis, disk herniation, and lumbar spondylolisthesis are generally required lumbar spinal fusion surgery. Lumbar spinal fusion surgery procedures were usually processed with the decompression, instrumentation, correction, and fusion procedures step by step [1]. Because the anatomical structures of the spine have spongy vertebrae with rich blood supply and fragile venous plexus, substantial blood loss frequently occurs during the PLIF and increases postoperative morbidity, and prolongs clinical recovery [2, 3]. Massive blood loss makes the patients suffer a higher risk of developing cardiopulmonary events, renal failure, and cerebral infarction, especially for aged patients (over 60 years old) [4, 5]. Although allogeneic blood transfusions could prevent the patients from suffering the above life-threatening complications, blood transfusions are usually limited with blood supply, potential risk of immunologic reaction, and infectious disease transmission [6]. Therefore, there is a need to find safe and effective methods to reduce blood loss in patients with PLIF.

There is a balance between coagulation and anticoagulation, fibrinolysis and antifibrinolysis in the human body. The coagulation system and fibrinolysis are activated almost simultaneously when the body is injured. Tranexamic acid (TXA) binds to the plasminogen and allows the plasminogen cannot be activated to plasmin to achieve hemostasis, which is currently the goal of the action of antifibrinolytic drugs [7]. TXA is a synthetic antifibrinolytic agent that inhibits the binding of plasminogen, plasmin, and tissue plasminogen activator by competing for lysine binding sites. Therefore, it can delay fibrinolysis and clot degradation to achieve the purpose of reducing intraoperative blood loss [8]. The drug exerts significant hemostatic effects in hip replacement and coronary artery surgery [9, 10]. Recently, new studies have shown that the use of TXA in patients undergoing scoliosis surgery results in a significant reduction in blood loss [11]. This study intends to collect relevant randomized controlled trials (RCT) to systematically evaluate the efficacy and safety of TXA in the treatment of lumbar degenerative diseases with PLIF.

## Methods

This meta-analysis followed the Cochrane handbook for conducting and the Preferred Reporting Items for Systematic Reviews and Meta-Analyses (PRISMA) guidelines for reporting [12, 13]. Two authors separately conducted literature retrieval, study eligibility, data extraction, and quality assessment with inconsistency solved by discussion and decided by the corresponding author.

### Literature search

Randomized controlled trials evaluating the efficacy and safety of tranexamic acid in posterior lumbar interbody fusion (PLIF) were searched by computer from the inception of the PubMed, Cochrane Library, Web of Science, and Embase databases until August 2022. The language searched was English, and the search terms included: posterior lumbar interbody fusion, lumbar degenerative disease, spinal surgery, tranexamic acid, and randomized controlled trial. The brief retrieval formula was “(tranexamic acid) AND (lumbar degenerative disease) AND (randomized controlled trial) AND ((spinal surgery)) OR (posterior lumbar interbody fusion))”.

### Inclusion and exclusion criteria

The inclusion criteria were as follows: randomized controlled trial (RCT) of intraoperative hemostasis with TXA in patients undergoing posterior lumbar interbody fusion (PLIF) for lumbar degenerative disease; intervention methods were TXA (intravenous TXA, local TXA, intravenous combined with local TXA) in the experimental group and placebo in the control group; patients were older than 18 years; results included at least two of the following indicators: total blood loss, intraoperative blood loss, postoperative drainage volume, operation time, number of blood transfusions, and deep venous thrombosis.

Exclusion criteria were as follows: no randomized controlled trial (RCT); no posterior lumbar interbody fusion (PLIF) surgical procedure; missing data or mistakenly unusable literature; literature on patients with severe liver and kidney function or tumors; literature on patients with coagulation abnormalities; review, meeting, expert opinion, case report, literature that could not obtain the full text; animal experiments, in vitro/biomechanical studies.

### Literature screening and data extraction

Two researchers independently screened the literature and extracted the data according to the inclusion and exclusion criteria, cross-checked the screening results, and discussed and solved the dispute with a third party. The first review of the titles and abstracts is to complete the primary screening, exclude the studies that are irrelevant, and read the full text to complete the rescreening if necessary. The main contents of extracted data include first author and publication time; study type; sample size; and outcome measures.

### Literature quality evaluation

The risk of bias in the included studies was independently evaluated by two authors and the results were cross-checked, with the disagreement being decided jointly by a third author. The original studies included were assessed based on different study designs and methodologies, using the Cochrane Handbook 5.1 recommended RCT Bias Risk Assessment Tool for randomized controlled trials.

### Statistical analysis

A meta-analysis of extracted data was performed using RevMan 5.4 software. The heterogeneity test was performed by the chi-square test, and the *I*^2^ value was calculated for quantification. If *P* ≥ 0.1 and *I*^2^ ≤ 50%, it indicated that there was homogeneity between studies, and a fixed-effects model was used; if *P* < 0.1 and *I*^2^ > 50%, it indicated that there was significant heterogeneity between studies, the random effect model was used, and mean difference (MD) was calculated for continuous variables; odds ratio (OR) was calculated for binary variables, and the point estimate and 95% confidence interval (CI) were given for each effect size. The test level was *P* = 0.05.

## Results

### Literature screening procedure and results

**Fig. 1:**
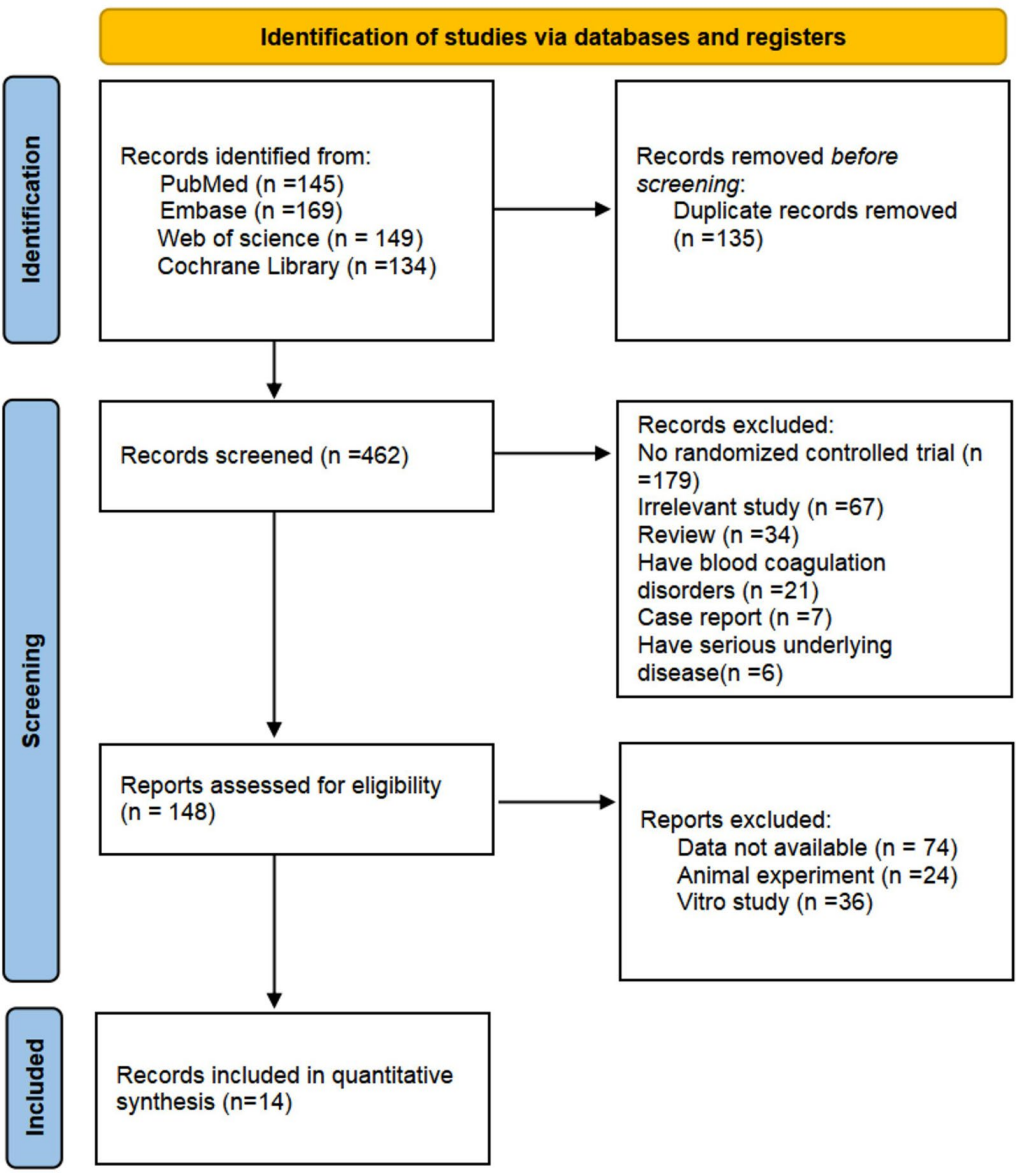
Flowchart illustrating the literature search and the selection of included studies

In this study, 597 papers were obtained through a preliminary search, 135 repeated publications were eliminated by software, titles and abstracts were read, and 314 papers that did not meet the inclusion criteria were eliminated. After careful reading of the full text and quality evaluation, 134 unqualified papers were further excluded, and 14 qualified papers [3, 14–26] were finally included. The paper screening process is presented in Fig. 1. Fourteen of them were high-quality RCTs. A total of 1681 patients were included, including 836 patients in the TAX group and 845 patients in the control group. The main characteristics of the included studies are presented in Table 1.

**Table 1:** Basic characteristics of the included studies

Study	Intervention measures	N (Male/Female)		Age (years)		Weight (kg)	
		TXA	CG	TXA	CG	TXA	CG
Elwatidy et al. [14]	30 mg/kg + 1 mg/(kg h) A + B	32 (21/11)	32 (18/14)	51.56 + 19.08	49.75 + 21.04	72.48 ± 13.81	69.63 ± 17.29
Wong et al. [15]	10 mg/kg + 1 mg/(kg h) A + B	73 (21/52)	74 (26/48)	56.8 ± 16.2	50.0 ± 16.2	72.9 ± 17.2	73.9 ± 16.1
Liang et al. [16]	2000 mg C	30 (15/15)	30 (14/16)	51.13 ± 10.72	53.50 ± 10.26	26.2 ± 4.1 (BMI)	25.3 ± 5.2 (BMI)
Shi et al. [17]	30 mg/kg + 2 mg/(kg h) A + B	50 (25/25)	46 (22/24)	53.76 ± 12.06	55.87 ± 13.14	63.10 ± 10.82	62.13 ± 9.98
Nagabhushan et al. [3]	10 mg/kg + 1 mg/(kg h) A + B	25 (9/16)	25 (11/14)	49.60 ± 9.79	51.72 ± 9.71	58.84 ± 3.31	60.96 ± 7.35
Kim et al. [18]	10 mg/kg + 2 mg/(kg h) A + B	24 (12/12)	24 (15/9)	61.0 ± 9.0	65.2 ± 7.0	25.9 ± 3.2 (BMI)	25.1 ± 3.2 (BMI)
Wang et al. [19]	10 mg/kg + 1 mg/(kg h) A + B	39 (25/18)	41 (18/19)	41.2 ± 10.3	42.5 ± 9.5	63.2 ± 18.3	64.3 ± 19.1
Mu et al. [20]	15 mg/kg A	45 (27/18)	42 (23/19)	54.20 ± 7.37	52.57 ± 6.73	24.83 ± 1.95 (BMI)	23.93 ± 1.35 (BMI)
Ou et al. [21]	15 mg/kg + 1 g A + C	59 (30/29)	59 (31/28)	64.2 ± 4.6	64.0 ± 5.1	22.64 ± 3.29 (BMI)	22.57 ± 3.20 (BMI)
Sun et al. [22]	15 mg/kg A	26 (15/11)	37 (22/15)	57.12 ± 11.55	51.51 ± 13.57	24.86 ± 4.72 (BMI)	24.44 ± 3.57 (BMI)
Xu et al. [23]	1 g C	30 (13/17)	30 (14/16)	49.6 ± 12.8	50.6 ± 16.2	25.3 ± 3.0 (BMI)	25.7 ± 3.0 (BMI)
Zhang et al. [24]	1 g + 1 g A + C	151 (57/94)	138 (47/91)	54.73 ± 9.93	57.04 ± 10.20	25.82 ± 3.28	25.20 ± 3.54
Li et al. [25]	2 g + 1 g A + C	212 (74/138)	227 (84/143)	55.4 ± 10.61	55.26 ± 10.44	25.73 ± 3.31 (BMI)	25.71 ± 3.63 (BMI)
Yan et al. [26]	20 mg/kg + 10 mg/(kg h) A + B	40 (16/24)	40 (22/18)	53 ± 7	52 ± 8	25.4 ± 3.1 (BMI)	24.4 ± 3.4 (BMI)

*A* Intravenous use; *B* Intraoperative maintenance; *C* Local use; *BMI* Body mass index; *CG* Control group; *TXA* Tranexamic acid group

### Quality analysis of included studies

**Fig. 2:**
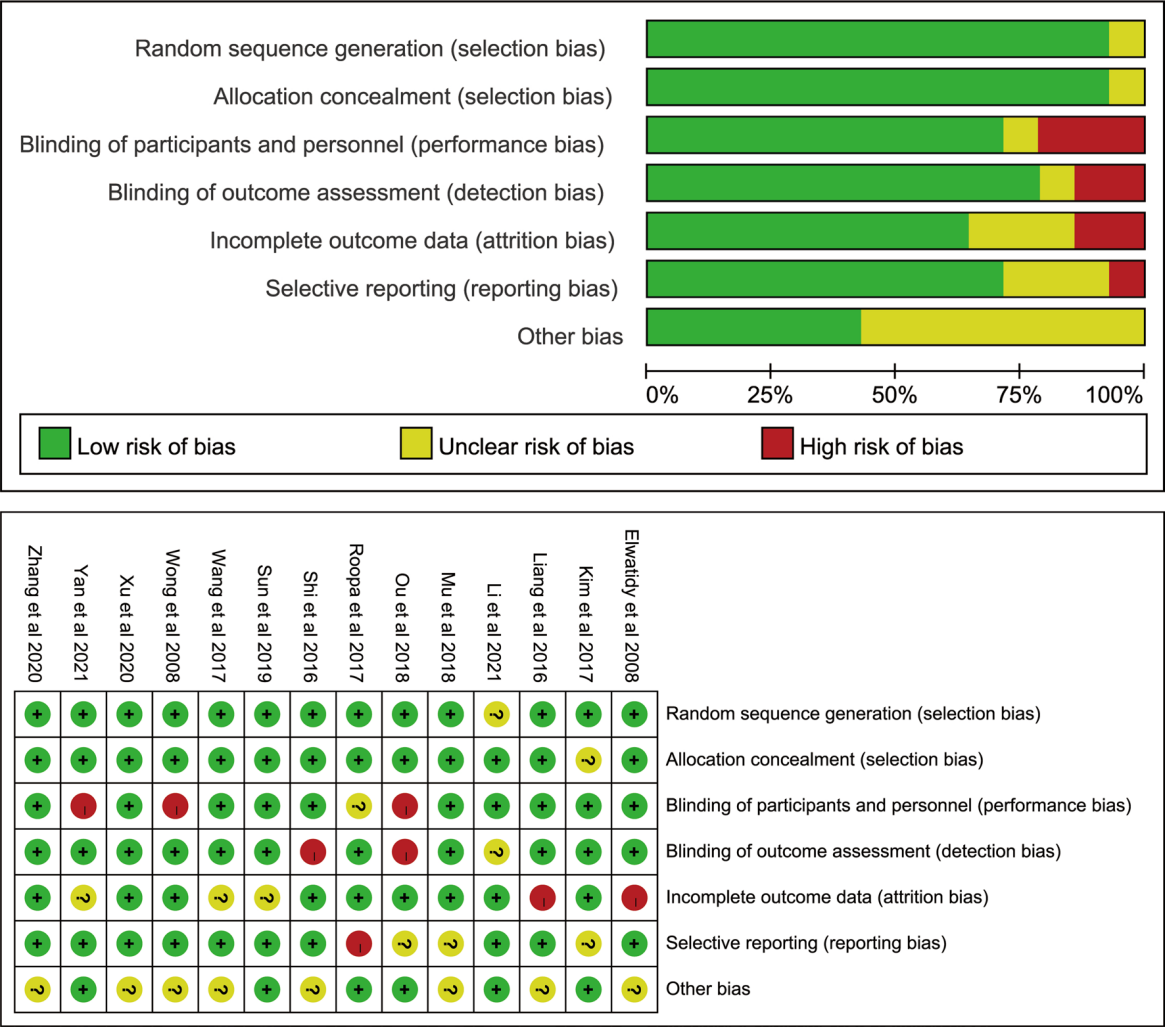
Risk-of-bias graph for each included study

For the fourteen included RCT studies, risk assessment was performed according to the Cochrane Risk of Bias tool, as shown in Fig. 2.

### Meta-analysis results

#### Intraoperative blood loss

**Fig. 3:**
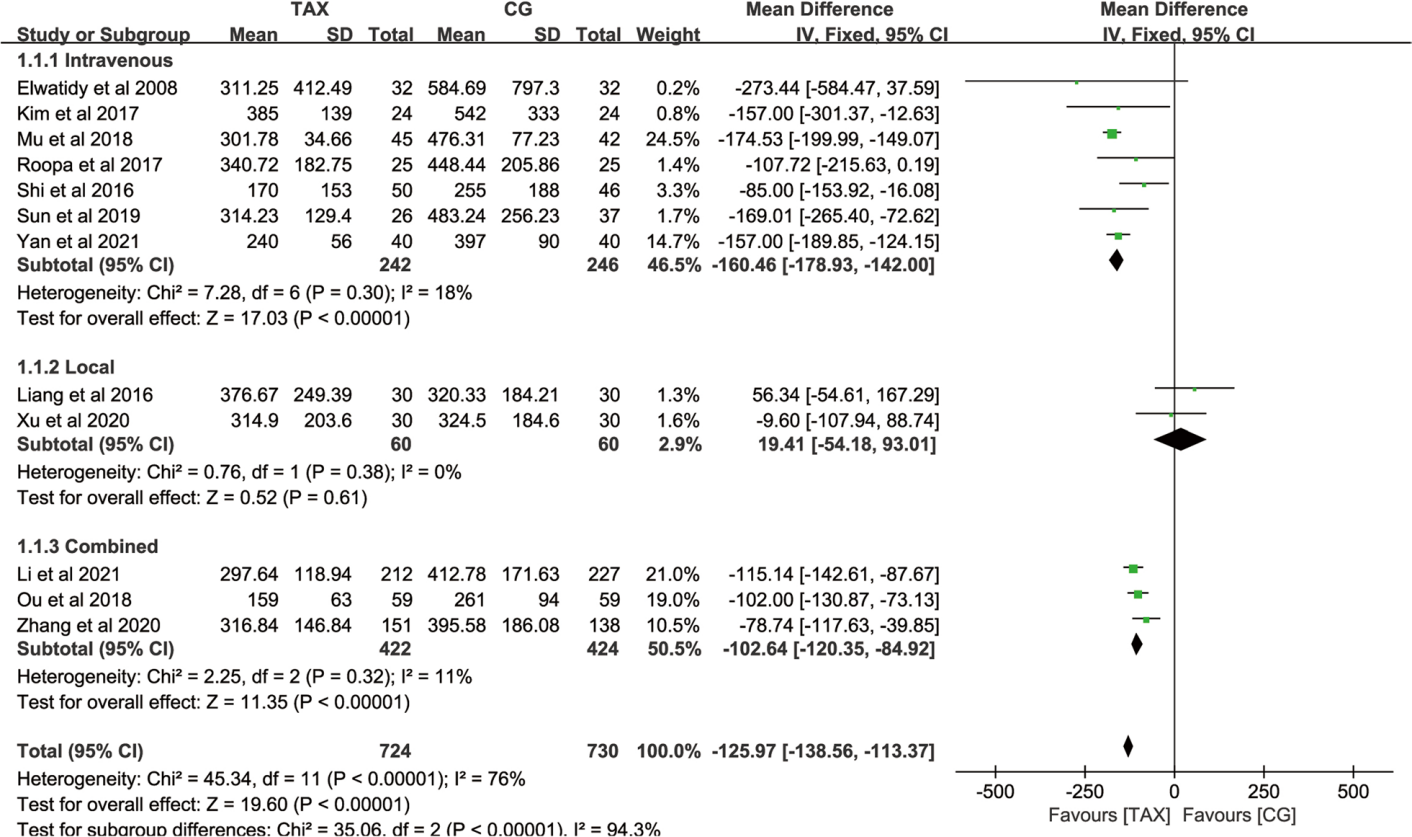
Forest plot of intraoperative blood loss

A total of 12 studies counted intraoperative blood loss. There was significant heterogeneity across studies (*P* = 0.0001, *I*^2^ = 76%), so subgroup analyses were performed. According to different methods of using TXA, each study was divided into intravenous TXA, local TXA, and combined TXA. The results of subgroup analysis showed no significant heterogeneity in the intravenous TXA subgroup (*P* = 0.30, *I*^2^ = 18%); local TXA subgroup (*P* = 0.38, *I*^2^ = 0%), and intravenous combined with the local TXA subgroup (*P* = 0.32, *I*^2^ = 11%), indicating that different methods of TXA utilization are responsible for significant heterogeneity between studies in intraoperative blood loss. Meta-analysis was performed using a fixed-effects model, and significant differences were observed in the intravenous TXA subgroup [MD = − 160.46, 95% CI (− 178.93, − 142.00), *P* < 0.0001] and the combined TXA subgroup [MD = − 102.64, 95% CI (− 120.35, − 84.92), *P* < 0.0001], indicating that the intravenous TXA and the combined TXA could reduce the intraoperative blood loss of patients [MD = − 125.97, 95% CI (− 138.56, − 113.37), *P* < 0.0001]. However, there was no statistical difference in the local TXA group [MD = 19.41, 95% CI (− 54.18, 93.01), *P* = 0.61], indicating that there was no significant effect on intraoperative blood loss because topical TXA was applied directly into the surgical incision after surgery (Fig. 3).

#### Total blood loss

**Fig. 4:**
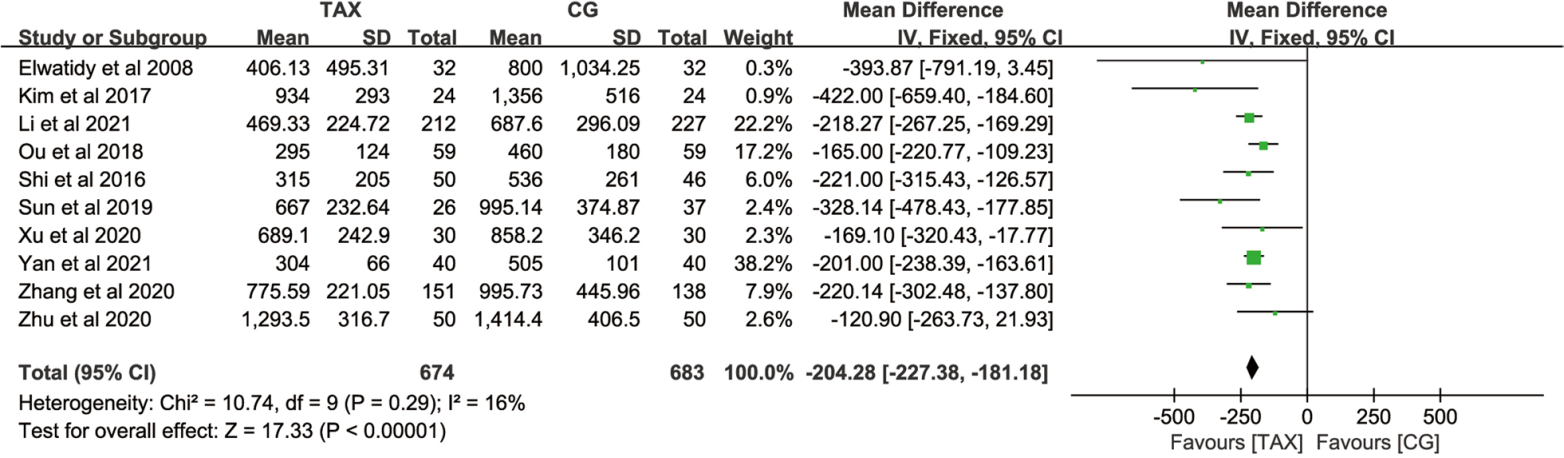
Forest map of total blood loss

Total blood loss was counted in 9 studies. A total of 1257 patients provided total blood loss data, with no significant heterogeneity across studies (*P* = 0.29, *I*^2^ = 16%), and a fixed-effects model was used for meta-analysis. The results showed that total blood loss in the TXA group was significantly lower than that in the control group [MD = − 204.28, 95% CI (− 227.38, − 181.18), *P* < 0.00001] (Fig. 4), indicating that TXA had a certain effect on the reduction in total blood loss in patients.

#### Postoperative drainage

**Fig. 5:**
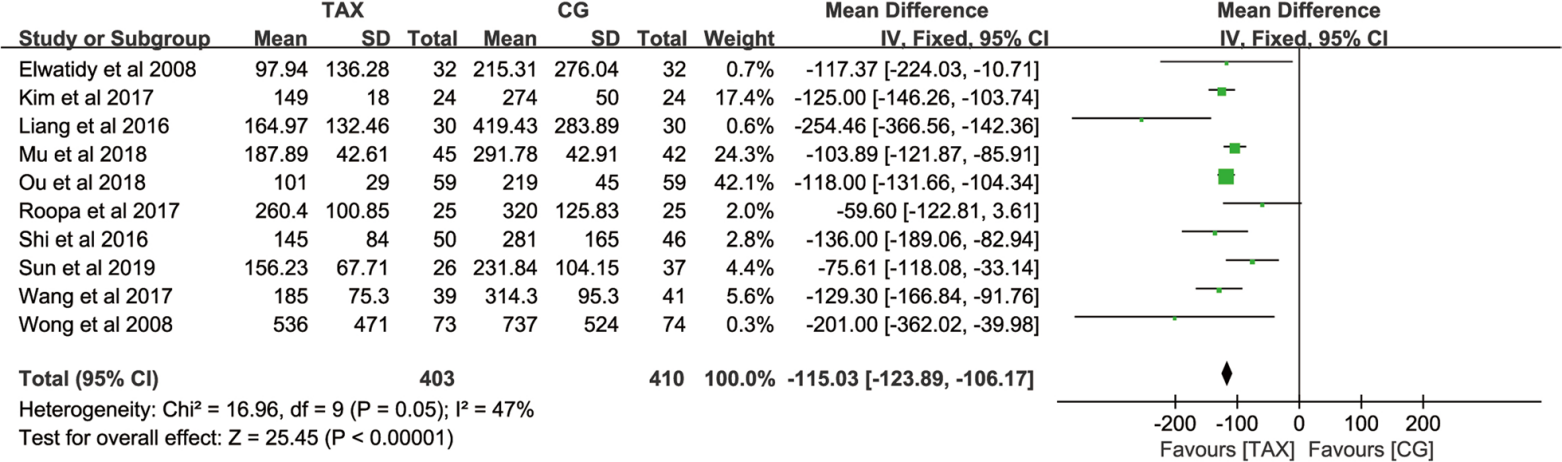
Forest map of postoperative drainage

Postoperative drainage was counted in 10 studies. A total of 813 patients provided total blood loss data, with no significant heterogeneity across studies (*P* = 0.05, *I*^2^ = 47%), and a fixed-effects model was used for meta-analysis. The results showed that postoperative drainage in the TXA group was significantly lower than that in the control group [MD = − 115.03, 95% CI (− 123.89, − 106.17), *P* < 0.00001] (Fig. 5), indicating that TXA had a certain effect on the reduction in postoperative drainage in patients.

#### Operation time

**Fig. 6:**
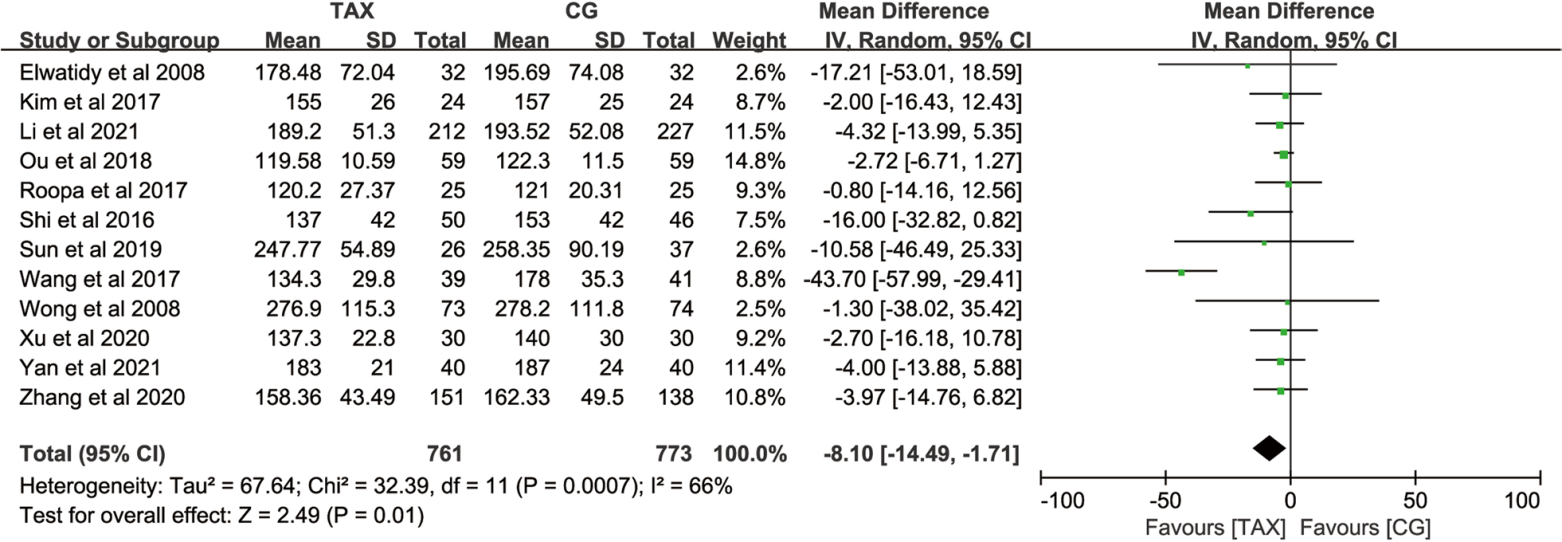
Forest map of operation time

A total of 9 studies used operation time as an outcome measure, with 761 patients in the TXA group and 773 patients in the control group. The heterogeneity test (*P* = 0.0007, *I*^2^ = 66%) suggested that there was significant heterogeneity between the studies, and a meta-analysis using a random-effects model showed that: [MD = − 8.10, 95% CI (− 14.49, − 1.71), *P* = 0.01] (Fig. 6), suggesting that the application of TXA during PLIF surgery could shorten the operation time, and the difference was statistically significant.

#### Transfusion rate

**Fig. 7:**
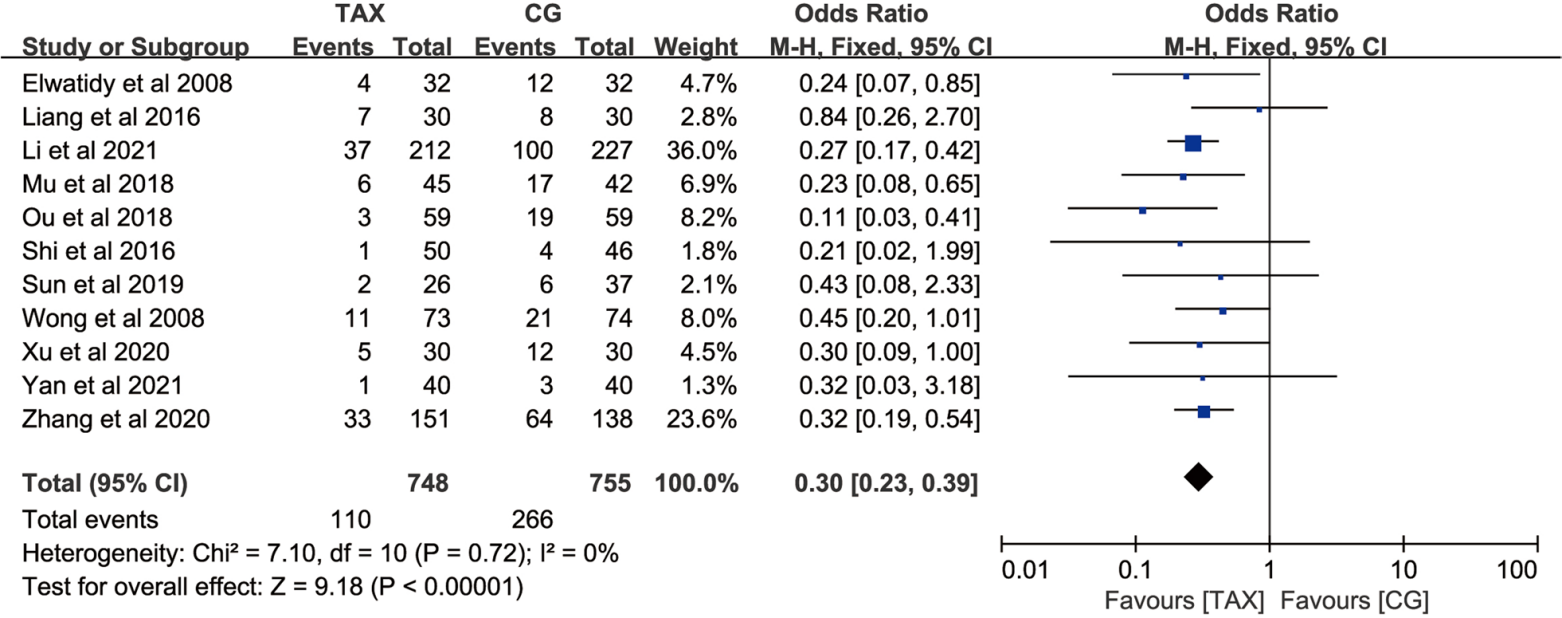
Forest map of the number of blood transfusion cases

A total of 11 studies used the incidence of transfusion events as an outcome measure, with 748 patients in the TXA group and 755 patients in the control group. The heterogeneity test (*P* = 0.72, *I*^2^ = 0%) suggested that there was homogeneity between the included studies, and a fixed-effects model was used for meta-analysis, which showed: [OR = 0.30, 95% CI (0.23, 0.39), *P* < 0.00001] (Fig. 7), suggesting that TXA has the effect of reducing perioperative blood transfusion in PLIF surgery, and the difference was statistically significant.

#### Incidence of deep venous thrombosis

**Fig. 8:**
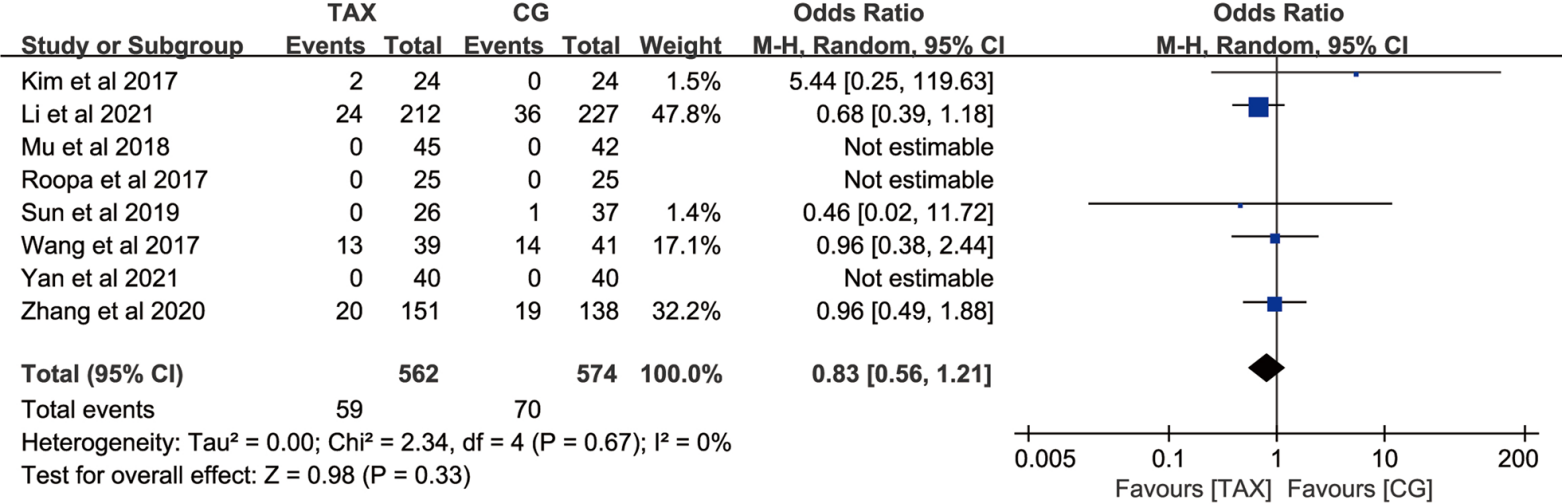
Forest map of the number of cases of deep vein thrombosis

A total of 8 studies used the incidence of transfusion events as an outcome measure, with 562 patients in the TXA group and 574 patients in the control group. In the heterogeneity test (*P* = 0.67, *I*^2^ = 0%), which suggested that there was homogeneity between the included studies, a fixed-effects model was used for meta-analysis, and the results showed: [OR = 0.83, 95% CI (0.56, 1.21), *P* = 0.33] (Fig. 8), suggesting that there was no significant difference in the incidence of deep venous thrombosis between TXA and placebo in the perioperative period of PLIF surgery.

### Heterogeneity and sensitivity analyses

The results of this analysis showed that the heterogeneity of intraoperative blood loss and operation time was high. The sensitivity analysis was performed after removing the included literature in the operation time turn. The analysis results were consistent with the conclusions before removing them, indicating that the heterogeneity had little effect on the results of this study. Heterogeneity may arise from the skill level of the operator. Removing Ou et al. [21] from intraoperative blood loss reduced heterogeneity by 21%, while removing other studies did not change significantly. The heterogeneity may be caused by the different experience levels of surgeons, recording methods of intraoperative blood loss, and incomplete medical records.

### Publication deviation

**Fig. 9:**
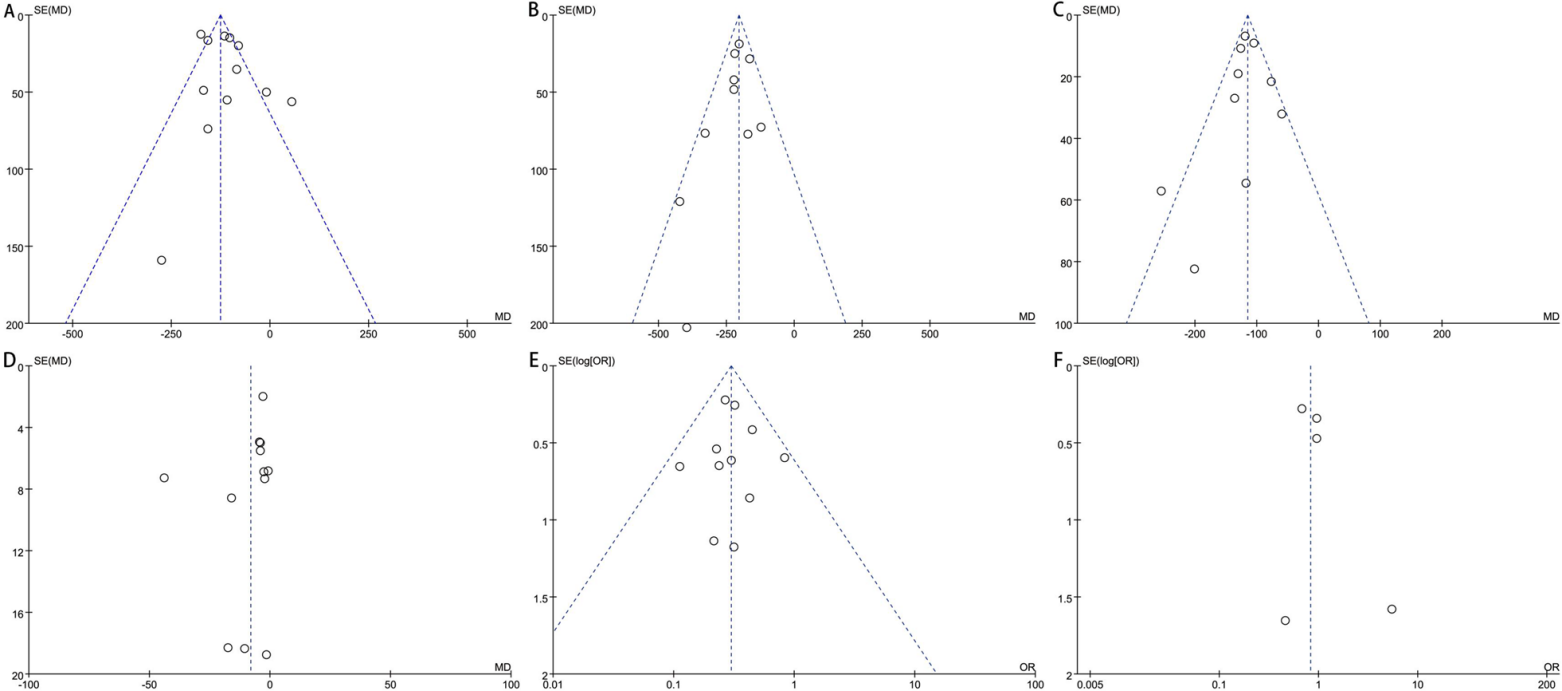
**A** Funnel plot of publication bias for intraoperative blood loss; **B** funnel plot of publication bias for total blood loss; **C** funnel plot of publication bias for postoperative drainage; **D** funnel plot of publication bias for operation time; **E** funnel plot of publication bias for the number of blood transfusion cases; **F** funnel plot of publication bias for the number of cases of deep vein thrombosis

A total of 14 articles were included in this study. All outcome measures were tested for publication bias. It could be seen that the funnel plot of each outcome measure was visually basically symmetrical, suggesting that there was a small possibility of publication bias (Fig. 9).

## Discussion

TXA is a synthetic lysine derivative with a high affinity to the lysine binding region of plasminogen and plasmin, which competitively inhibits the lysine binding site of fibrin from binding to plasminogen, and prevents plasmin from degrading fibrin to achieve hemostasis [27]. It has been used in various clinical treatments, including oral surgery, gynecological surgery, upper gastrointestinal bleeding, cardiac surgery, postpartum hemorrhage, etc., and has been widely recognized. In the view of spinal anatomy, it is usually not easy to reduce the blood loss of cancellous bone since the sufficient blood circulation. In addition, many hemostatic methods are limited by the procedures of spinal surgery, resulting in more intraoperative blood loss. Spinal surgery can cause extreme hypoxia after massive blood loss and even lead to shock and major organ arrest, so to ensure that patients are safely through the perioperative period, an allogeneic blood transfusion is often required. However, there are many risks associated with blood transfusion, such as fever, infection, transfusion reactions, transfusion-related infectious diseases, etc. [28, 29]. Therefore, how to reduce intraoperative and postoperative bleeding and oozing will certainly become a very concerning issue for orthopedic surgeons and has very important clinical significance. It has been shown that TXA is effective and safe in reducing blood loss in spinal surgery [18, 20, 22, 25, 26].

Intraoperative blood loss directly affects the operation time and clear surgical field, so it has a significant impact on the operation. However, it remains unclear whether local use of TXA can significantly reduce intraoperative blood loss in patients with PLIF. Liang et al. [16] showed that the amount of bleeding in the gelatin foam group soaked with TXA in patients with PLIF was similar to that in the gelatin foam group. Ren et al. [30] divided 100 patients who needed lumbar surgery into two groups, the TXA local application group (1 g TXA dissolved in 100 ml normal saline soaked for 5 min before closing the incision) and the control group (equal amount of saline). The results showed that patients in TXA local application group had reduced postoperative bleeding volume, and significantly shortened drainage tube removal time and hospital stay. Shi et al. [31] divided 120 patients scheduled for posterior thoracolumbar fusion into two groups: local TXA group (the wound surface was topically soaked with TXA (1 g in 100 mL of saline solution) for 5 min before wound closure) and control group. The study results showed that the local TXA group had significantly less postoperative blood transfusion volume and postoperative drainage volume than the control group, and no complications occurred during the late follow-up. It was confirmed that TXA can be absorbed into the blood to exert a hemostatic effect when applied topically, so it was considered whether TXA can also be absorbed into the blood locally to exert a hemostatic effect when applied topically during PLIF. It remains unclear whether intravenous injection combined with topical TXA is superior to TXA alone. A meta-analysis by Sun et al. [32] showed that intravenous combined with local application of TXA was superior to intravenous TXA alone in patients undergoing total hip arthroplasty in terms of blood loss (hemoglobin drop and transfusion rate). Ou et al. [21] showed that combined intravenous and local application of TXA (intravenous application: 15 mg/kg; local application: 1 g TXA dissolved in 10 ml normal saline, fully soaked in 4 pieces of absorbable gelatin sponge placed in the incision) reduced blood transfusion rate and blood loss in two-level posterior lumbar decompression and fusion surgery without adverse effects. Therefore, it is of some interest to consider the combined use of TXA in patients with PLIF.

Although TXA has been widely used in the perioperative period of orthopedic surgery, there is a lack of corresponding standards of use, usually based on the surgeon's clinical experience. Low-dose administration usually does not achieve hemostasis well, whereas high-dose administration increases the risk of deep vein thrombosis. Kim et al. [18] showed that high-dose application of TXA (10 mg/kg intravenous maximum loading dose and 2 mg/kg/h infusion until skin closure) was more effective than low-dose TXA in reducing blood loss in patients with PLIF. The study by Xie et al. [33] suggests that high doses of TXA (100 mg/kg intravenous maximum loading dose and 10 mg/kg/h infusion until skin closure) can effectively control blood loss and reduce allogeneic blood transfusion without adverse drug reactions to spinal corrective surgery. Wang et al. [34] found that the transfusion rate decreased from 26.3% to 2.4% in the 15 mg/kg TXA group, whereas the 10 mg/kg TXA group did not significantly reduce the transfusion rate by reducing bleeding alone. However, the doses of TXA used intraoperatively and postoperatively varied among the included studies, and conclusions need further discussion.

The majority of patients undergoing spinal surgery are elderly, have perennial mobility difficulties, and even stay in bed for a long time, significantly increasing the risk of venous thrombosis [35]. Therefore, the use of hemostatic drugs in the perioperative period of spinal surgery must consider whether there is an increased risk of venous thrombosis. Sun et al. [22] retrospectively reviewed the clinical data of 63 patients who underwent 1–3 level posterior lumbar interbody fusion and received intravenous TXA (*n* = 26) and normal saline as placebo (*n* = 37) during surgery and concluded that prophylactic intravenous tranexamic acid 30 min before intraoperative skin incision effectively reduced perioperative blood loss, drain indwelling time, and hospital stay without increasing complications. Lin et al. [36] noted that high doses (50 mg/kg intravenous maximum loading dose and 5 mg/kg/h infusion until skin closure) were effective in reducing intraoperative bleeding in complex adult spinal deformity surgery, but there were three thromboembolic complications after surgery. Therefore, the dose of TXA should be determined by the type of spinal surgery, the patient's condition (e.g., weight, renal function), and other factors. In addition, an associated dose–response analysis should be performed to determine the optimal TXA dose in spinal surgery. Obtaining sufficient antifibrinolytic enzyme to inhibit bleeding in the surgical area using TXA without side effects is the most ideal therapeutic effect that TXA can achieve.

Spinal surgery for the multilevel disease is inevitable due to its long duration, invasiveness, and blood loss. Excessive blood loss may increase the risk of infection, intraspinal hematoma, and transfusion, which may lead to an immune response or viral transmission. How to reduce perioperative blood loss and blood transfusion rate in spinal surgery has become the focus of research. TXA is a synthetic antifibrinolytic agent that has been widely used to reduce perioperative blood loss in spinal surgery. Intravenous or local application of TXA was effective in reducing perioperative blood loss and transfusion rates in spinal surgery, with no significant increase in the risk of venous thrombosis. Therefore, the application of TXA in the perioperative period of spinal surgery is feasible. However, there is no uniform standard for the route, dose, timing of administration of TXA, and mode of use to improve safety. For spinal surgery, postoperative bleeding may increase the formation of epidural hematomas, causing neurological disorders. Therefore, whether TXA should be continued postoperatively in patients with suspected bleeding warrants further investigation. Most previous studies excluded patients with severe heart and lung disease. However, it is important for these patients to reduce perioperative blood loss, so how to use TXA in such patients should be further explored in future spine surgery-related studies.

This study also has shortcomings: (1) Because it is difficult to achieve complete implementation of randomized control and blinding in practical work, some studies do not mention the specific methods of allocation concealment and blinding operation in the study, so there may be selection bias in the selection and allocation of patients, affecting the quality of the included literature; (2) there is a lack of guidelines for the clinical use of TXA, and the dose and mode of use of TXA are different in the included studies, resulting in high heterogeneity of some combined results; (3) the method of use and dose of TXA described in this study are not distinguished, which may increase the bias of the article; and (4) most literature works in this study are small samples, which will have a certain impact on the reliability of the results and require more large-sample multicenter RCTs to verify the conclusions.

## Conclusions

In conclusion, based on the current meta-analysis results, tranexamic acid can be considered safe and effective in posterior lumbar interbody fusion and is superior to the control group in shortening the operation time, reducing intraoperative blood loss and postoperative drainage volume, total blood loss, and blood transfusion rate, but has no significant advantage in the incidence of deep venous thrombosis compared with the control group.

## Data Availability

The data sets generated and analyzed during the current study are not publicly available but can be obtained from the corresponding author upon reasonable request.

## Abbreviations

CI: Confidence interval
DVT: Deep vein thrombosis
MD: Mean difference
OR: Odds ratio
PLIF: Posterior lumbar interbody fusion
RCT: Randomized controlled trial
TXA: Tranexamic acid
